# Underground Coal Mining: Relationship between Coal Dust Levels and Pneumoconiosis, in Two Regions of Colombia, 2014

**DOI:** 10.1155/2015/647878

**Published:** 2015-08-20

**Authors:** Carlos Humberto Torres Rey, Milciades Ibañez Pinilla, Leonardo Briceño Ayala, Diana Milena Checa Guerrero, Gloria Morgan Torres, Helena Groot de Restrepo, Marcela Varona Uribe

**Affiliations:** ^1^Universidad del Rosario, Bogotá, Colombia; ^2^Instituto Nacional de Salud, Bogotá, Colombia; ^3^Positiva ARL, Bogotá, Colombia; ^4^Universidad de Los Andes, Bogotá, Colombia

## Abstract

In Colombia, coal miner pneumoconiosis is considered a public health problem due to its irreversibility, high cost on diagnosis, and lack of data related to its prevalence in the country. Therefore, a cross-sectional study was carried out in order to determine the prevalence of pneumoconiosis in underground coal mining workers in two regions of Colombia. The results showed a 35.9% prevalence of pneumoconiosis in the study group (42.3% in region 1 and 29.9% in region 2). An association was found between a radiologic diagnosis of pneumoconiosis and a medium risk level of exposure to carbon dust (OR: 2.901, 95% CI: 0.937, 8.982), medium size companies (OR: 2.301, 95% CI: 1.260–4.201), length of mining work greater than 25 years (OR: 3.222, 95% CI: 1.806–5.748), and a history of smoking for more than one year (OR: 1.479, 95% CI: 0.938–2.334). These results establish the need to generate an intervention strategy aimed at preventing the identified factors, as well as a timely identification and effective treatment of pneumoconiosis in coal miners, in which the commitment of the General Health and Social Security System and the workers compensation system is ensured.

## 1. Introduction

Coal is a combustible, carbonaceous, sedimentary rock composed mostly of carbon and hydrocarbons. It is a fossil fuel used in processes such as the production of energy and iron, cement manufacturing, and other industrial processes [1, 2]. Based on the characteristics of carbon content, percentage of noncombustible minerals, moisture, and calorific power, the varieties of coal are classified into four different types or ranking levels of coal, each with differences in energy output as a result of increased pressurization, heat, and time. The four types are anthracite or hard coal, bituminous or fatty coal, subbituminous or black lignite, and lignite-peat [1]. The coal forms with greater combustion capacity have the greatest risk of causing coal miner pneumoconiosis (CMP), due to having the most surface free radicals [3, 4].

Coal mine dust is a complex and heterogeneous mixture containing more than 50 different elements that include carbon, crystal silica, and other trace elements such as boron, cadmium, nickel, iron, antimony, lead, and zinc, among others [5]. Coal worker's pneumoconiosis (CWP), a nodular interstitial lung disease that, in severe cases, may lead to progressive massive fibrosis (PMF) [6], is the work related disease most frequently associated with coal mining [7–9] and also is a serious occupational disease worldwide, especially in developing countries [10–13]. It is produced as a result of tissue reaction due to the accumulation of inhaled coal dust in the lungs; it is a chronic condition which develops slowly (it usually takes at least ten years to show signs), and, due to its progressive and irreversible nature, it causes a great economic and social impact. Therefore, Colombia, a coal producing and exporting country, in an effort to prevent and eliminate CWP, developed the “national plan for preventing silicosis, coal worker's pneumoconiosis, and asbestosis” which seeks to improve the quality of life of exposed workers and their families, as well as to improve the competitiveness of the companies where exposure to these agents occurs [14].

The risk of developing the illness is a function of the degree of accumulated exposure to coal dust throughout the work life [5, 6, 15]. A study of pneumoconiosis and massive pulmonary fibrosis in 6,658 mine workers from 416 mines in 15 states in the United States, between 2005 and 2009, determined that the prevalence of pneumoconiosis in underground coal mine workers varied between 4.8 and 9% [16]. In Colombia, in 2000, in the department of Antioquia, a study was carried out addressing the pneumoconiosis situation in coal miners, in which 189 cases of pneumoconiosis were found [17]. Another study carried out to determine the prevalence of pneumoconiosis in other regions of Colombia concluded that the prevalence was near 5% (95% CI: 2.6–7.64 × 10^2^), which contrasted with the national prevalence data found in 1988 which was near 2% [18]. In the 2003–2005 Diagnostic Report of Professional Diseases in Colombia, within the pulmonary diseases reported by the workers compensation insurance agencies in 2005, asthma and pneumoconiosis contributed between 3% and 4% to all the work related illnesses [19]. However, current data are not available in Colombia to determine the magnitude of the problem and the associated factors. This study, in 2013 and 2014, determined the prevalence of pneumoconiosis in coal miners in the departments of Boyacá and Cundinamarca and the relationship to environmental levels of coal and silica dust and other factors such as the length of time performing underground mining activities (duration of exposure) and, with these results, provides for the development of effective interventions in underground mine workers.

## 2. Materials and Methods

A cross-sectional study was carried out between 2013 and 2014 in twenty-nine companies affiliated with a workers' compensation insurance company, involved in underground coal mining, of which eighteen (18) were located in the department of Boyacá and eleven (11) in Cundinamarca. The criteria used for worker inclusion were age over 18 years old, 10 or more years working in the mining sector, employed by the selected company at the time of the field work, and voluntary acceptance to participate in the study. Workers were excluded from the study if they had a prior diagnosis of tuberculosis and/or had a condition which would contraindicate the use of forced spirometry.

In Colombia all employers must register their workers with an insurance company in order to assure the welfare and economic benefits derived from work accidents or an occupational disease. The population of this study was a list of 466 companies with a total of 14,378 members of the State Insurance Company, which brings together the largest number of companies engaged in underground coal mining workers in the country. The study companies were selected by random, probabilistic, two-stage (primary sampling unit companies and subunit worker) sampling, stratified by department (Boyacá and Cundinamarca) and company size (small: under 50 employees, medium: 50 to 99 employees, and large: 100 or more employees) in conglomerates (companies) with proportional allocation. The sample was composed of 447 workers (232 from Boyacá and 215 from Cundinamarca) who were also selected randomly (Table 1).

Three instruments were designed for data collection. The first, entitled “survey for evaluating the work environment,” which was applied to the company by an environmental administrator specialized in Hygiene and Occupational Health, and which characterized exposure, established groups of similar exposure (GSE), and identified those workers who would receive the breathing air sampling equipment (GIL AIR PLUS pump with its respective sampling train). The collected samples were sent to an Industrial Hygiene and Toxicology Laboratory, where the air concentration of the respirable fraction of bituminous coal in coal dust (NIOSH 0600 method “Determination of MPFR Respirable Dust,” analytical technique: gravimetry) and crystalline silica levels (NIOSH 7602 method “Determination of Crystalline Silica by IR”, technique: infrared absorption spectrophotometry) was determined. For bituminous coal in coal dust, the reference criterion used was the maximum allowable level established by the American Conference of Governmental Industrial Hygienists (ACGIH), corrected for 8 hours of work six days a week, of 0.70 mg/m^3^. Four risk levels were set: low (obtained concentration/corrected TLV ratio less than 0.5), medium (ratio from 0.5 to 1), high (greater than 1 but less than 5), and severe (ratio greater than 5). For crystalline silica, the reference criterion used was the maximum allowable level established by the American Conference of Governmental Industrial Hygienists (ACGIH), corrected for 8 hours of work six days a week, of 0.02 mg/m^3^. Five risk levels were set: low (obtained concentration/corrected TLV ratio less than 0.5), medium (ratio from 0.5 to 1), high (greater than 1 but less than 2), severe (ratio from 2 to 5), and critical (ratio greater than 5).

The second instrument was the “worker's survey,” which was applied directly to the worker by a professional chemist who had been previously trained. Information was gathered regarding the worker's social and demographic characteristics (age, sex, schooling, place of residence, socioeconomic strata, educational level, and marital status), occupational characteristics (length of time engaged in mining activities, jobs held, and job and length of exposure at the time of the fieldwork), toxicological characteristics (smoking and alcohol consumption), and information related to respiratory symptoms. The third instrument was the “Occupational Medical History” which recorded information from the occupational medical evaluation performed by medical specialists with a current license to practice occupational health. In addition, the workers who underwent occupational health evaluation also performed forced spirometry under the direction of a respiratory therapist. Spirometries were made ensuring compliance with the criteria established by the American Thoracic Society (ATS), and, using the FEV1% value as a reference, five levels of severity of airflow limitation were set: mild (>70%), moderate (60–69%), moderately severe (50–59%), severe (35–49%), and very severe (<35%). A tuberculin test (which was administered by an occupational health professional), a chest X-ray (which was taken and read according to the requirements of the OIT technical guide for the OIT/2000 international classification of neumoconiosis), and X-rays (including reading by an NIOSH certified reader) were also performed. Workers were considered as cases of pneumoconiosis if the report of the ILO reader was “parenchymal abnormalities consistent with pneumoconiosis” (parenchymal abnormalities include small opacity profusion greater than or equal to 1/0 or large opacities). Workers who were reported by the ILO reader as unreliable or who required new chest radiography were classified, for the purposes of this paper, as negative for pneumoconiosis. Prior to data collection and performing medical and paraclinical assessments, each worker voluntarily agreed to participate in the study (this activity was documented through signing of the informed consent), with full assurance of the confidentiality of the information provided to the investigators.

Prior to beginning the fieldwork, a pilot study was performed which identified components of the instruments and of the application technique which were difficult for participants to understand and questions which caused confusion and/or poor interpretation; verified the degree of education and training of the interviewers selected to carry out the fieldwork; determined the time and movements needed for the application of the instruments and tests to be used; and established the optimal distribution of resources for carrying out the work. Likewise, prior to the field visit, each company was contacted by a team member in order to provide timely and sufficient information regarding the objectives and benefits of participating in the study and the mechanisms for ensuring the confidentiality of each company's information.

With regard to data systematization, a database was first constructed in Excel 97–2004 for Windows, and then it was purged to guarantee the quality of the information and avoid classification biases. 35% of the questionnaires were randomly reviewed, and wherever an error was found, all the questionnaires were reviewed. Finally, the information was processed using the SPSS version 22.0 package.

Keeping in mind the Ministry of Health Resolution 8430 of 1993 which establishes the academic, technical, and administrative norms regarding ethical aspects of research with human subjects, this study was classified as minimal risk and received approval from the Research and Ethics committees of the (National Institute of Health).

### 2.1. Statistical Analysis

The qualitative variables were described using distributions of absolute frequencies and percentages. For quantitative variables, measures of central tendency were calculated (average and median), as well as measures of dispersion (range and standard deviation).

The evaluation of the association between the categories of risk levels for coal dust, silica, and other factors and pneumoconiosis was determined using Pearson's Chi-square test of association or Fisher's exact test (expected values <5) and the OR and its respective 95% confidence interval. In order to evaluate the numeric scale of coal and crystal silica the assumptions of normality were evaluated with the Shapiro-Wilk test and the homogeneity of variance with Levene's test. Due to nonnormal distributions, the nonparametric Mann-Whitney and Kruskal-Wallis tests were performed. In order to determine the association between the level of risk of bituminous coal and pneumoconiosis, a multivariate analysis was performed, controlling for occupational variables and demographics using an unconditional logistic regression model. The precision of the estimators was evaluated using standard error and relative standard error. The statistical tests were evaluated at a 5% (*p* < 0.05) level of significance.

## 3. Results

Of the 29 underground coal mining companies selected, 18 (13 small, 2 medium, and 3 large) were located in the Boyacá department and 11 (3 small, 5 medium, and 3 large) in Cundinamarca. The size of the company, in order by frequency, was small 55.2% (*n* = 16), medium 24.1% (*n* = 7), and large 20.7% (*n* = 6). The sample was made up of 447 workers (446 men), with an average age of 43.1 ± 10.2 years and a range of 20 to 76 years, distributed in Cundinamarca (*n* = 215) and Boyacá (*n* = 232).

The workers' group was predominantly between the ages of 40 and 49, lived in a rural area, had a level 2 socioeconomic strata, had a permanent partner, and had finished elementary school. There were statistically significant differences by age group (*p* < 0.001), place of residence (*p* < 0.001), and socioeconomic strata (*p* < 0.001) and there was no significant difference by educational level (*p* = 0.273) and marital status (*p* = 0.511) (Table 2).

The average length of time in mining work was 19.49 ± 9.12 years with a median of 16 years; in Boyacá it was 17.43 ± 7.78 years (median 15) and in Cundinamarca it was 21.7 ± 9.99 years (median 20) (*p* < 0.001).

The prevalence of pneumoconiosis in workers was 35.87% (95% CI: 31.141%, 40.34%); in Cundinamarca it was 42.33% (95% CI: 35.67%, 48.98%), significantly greater than in Boyacá with 29.87% (95% CI: 23.92%, 35.82%) (*p* = 0.006). The most frequently observed parenchymal abnormalities were the q/q type (35.16% Cundinamarca, 44.93% Boyacá) corresponding to regular, small opacities between 1.5 cm and 3 cm in diameter. A worker in Boyacá presented small opacities associated with type A opacities, while in Cundinamarca four workers had large opacities (2 opacities associated with type A and 2 with type B) (Table 3).

In workers with less than 25 years of experience, the prevalence of pneumoconiosis was significantly lower than those who had 25–29.9 years and ≥30 years (26.5%, 57.1%, and 56.0%, *p* < 0.001), with similar results in Cundinamarca (26.6%, 65.7%, and 62.5%, *p* < 0.001), and they were not significant in Boyacá (26.4%, 42.9%, and 42.9%, *p* = 0.082).

In Cundinamarca, among the 5 GSE, significant differences were found in the prevalence of pneumoconiosis in development and tunnel advancement (47.4%, *n* = 74), transport (33.3%, *n* = 4), loading point (38.5%, *n* = 5), maintenance (17.5%, *n* = 5), and services (60.0%, *n* = 3) (*p* = 0.032). No significant differences were found in Boyacá (*p* = 0.098).

The environmental monitoring of coal dust reported an average concentration of bituminous coal of 3.27 ± 2.89 mg/m^3^ (median 3.12): 3.45 ± 3.42 mg/m^3^ (median 3.12) in Boyacá and 3.05 ± 2.11 mg/m^3^ (median 3.28) in Cundinamarca (*p* = 0.660).

In Boyacá, the levels of exposure to coal (*p* = 0.002) and silica (*p* = 0.004) of workers with pneumoconiosis were significantly higher than those of workers without pneumoconiosis. In Cundinamarca, no significant differences were found (*p* = 0.421 and *p* = 0.418). In Boyacá, there was a significant tendency of greater exposure to bituminous coal with pneumoconiosis (Table 4).

The prevalence of smoking for more than one year was 38.9% (Cundinamarca 37.7% and Boyacá 40.1%) and in Cundinamarca there was a greater prevalence of pneumoconiosis in workers who had smoked for more than one year (54.3% versus 35.1%, *p* = 0.004).

With regards to respiratory symptoms, 69.8% (*n* = 312) reported morning expectoration, 56.5% (*n* = 269) stated they had episodes of chronic cough during the day or night, 45.9% (*n* = 205) of workers reported that work had at some time caused a feeling of chest pressure, and 7.4% (*n* = 33) stated that they had not worked on at least one occasion due to respiratory problems. No statistically significant differences were found between the symptoms reported by the workers and the prevalence of pneumoconiosis.

Of the 447 spirometries performed, 89.11% (*n* = 398) were reported as normal, 5.11% (*n* = 23) showed findings compatible with peripheral airway alterations, 2.9% (*n* = 13) showed an obstructive pattern, 1.11% (*n* = 5) a restrictive pattern, 0.7% (*n* = 3) a mixed pattern, and 1.1% (*n* = 5) were discarded due to poor effort by the workers. No statistically significant differences were found between the results of the spirometry and the radiologic diagnosis of pneumoconiosis (*p* > 0.05).

Of the 439 readings of the tuberculin test, 77.9% (*n* = 342) were reported as negative, 8.2% (*n* = 36) as intermediate, and 13.9% (*n* = 61) with greater induration. No statistically significant differences were found between the tuberculin test results and the prevalence of pneumoconiosis in Cundinamarca (*p* = 0.419) and Boyacá (*p* = 0.451).

### 3.1. Multivariate Analysis

In the logistic regression model the factors that were found to be significantly associated with pneumoconiosis were medium level of exposure to coal dust, work history greater than 25 years in underground mining, and medium size companies; no differences were found by department or with smoking for more than one year. The level of exposure to silica was excluded from the final model because it did not show a significant association with pneumoconiosis (Table 5).

## 4. Discussion

Colombia possesses the greatest coal reserves in Latin America and its coal is known worldwide for having a low ash and sulfur content and having high volatile content and calorific power [20]. Coal, within the national mining production, has been the mineral which has shown a constant growth and the greatest variation beginning in 1990 and reaching a production of 85 million tons in 2011, of which 77 million (90.01%) were produced from open pit mining and 8 (9.9%) from underground mining [21, 22].

Of the 29 companies in the sample, 16 were small companies. These concentrated 50.6% of the evaluated workers, which coincides with the fact that the most prevalent mining in Boyacá and Cundinamarca departments is underground mining, which is carried out using moderately technified technology, on a small scale, and very often using artisanal methods. The distinctive coal development and production methods of underground mining create work environments which favor the development of health alterations especially associated with the respiratory system, which was corroborated by the fact that the evaluated companies had a higher frequency of risk levels. The fact that the middle sized companies in the study are mostly companies that are in a growing stage, are changing their production methods from manual to mechanized, and have not yet implemented all the intervention measures aimed at reducing the coal dust levels of silica in the workplace could explain why medium size companies had a higher risk of pneumoconiosis.

The observed values of coal dust and silica levels exceed the threshold levels. These high levels of exposure (coal dust >0.70 mg/m^3^ and silica: >0.02 mg/m^3^ for 8 hours of work, six days a week) increase the possibility of developing pneumoconiosis, so every effort needs to be made to reduce exposures both to respirable coal mine dust and to respirable crystalline silica.

The prevalence of pneumoconiosis in the study population was 35.9% (42.3% in Cundinamarca and 29.9% in Boyacá), much higher than that reported in previous studies carried out in the country [17, 18, 23]. However, although the increase in prevalence of pneumoconiosis coincides with that reported in other studies [13, 24, 25], we must take into account that the observed prevalence was evaluated in workers with more than 10 years of exposure to the risk factor, and pneumoconiosis is a dose-response-length of exposure to disease. The factors associated with pneumoconiosis in these workers were medium level of exposure, more than 25 years working in underground mining, and medium sized companies.

In this study, the prevalence of respiratory symptoms, especially those associated with morning expectoration and chronic cough, was very high in the study population (69.8% and 58.4%). Unlike a study carried out in a mine in Tanzania [26], the present study found no association between the prevalence of respiratory symptoms and the radiologic diagnosis of pneumoconiosis. However, with regard to respiratory symptoms reported by workers, statistically significant differences were found for critical levels of silica with chest tightness (*p* = 0.009) and shortness of breath (*p* = 0.05).

Some studies have reported a relationship between CWP and tuberculosis [13, 27–29]. Although the tuberculin test is useful for detecting latent and active tuberculosis, in the current study no statistically significant differences were found between the test results and the radiologic diagnosis of pneumoconiosis.

Spirometry evaluates the type of alteration and the severity of pulmonary lesions. The patterns observed in the current study, especially the high frequency of normal results, are consistent with reports in the literature that spirometry is only affected significantly when the extent of the disease increases [30–32].

All workers classified as “cases” were sent to the workers' compensation insurance companies to begin the process of diagnosis confirmation, according to protocols established in Colombia.

Finally, it is important to note that the workers' compensation insurance company, with which workers of this study were affiliated, has been at the forefront of the development and participation in research projects in order to increase awareness and generate intervention strategies that promote health and prevent occupational diseases.

## 5. Conclusions and Recommendations

The prevalence of pneumoconiosis in the study population (35.9%), much higher than previous studies, corroborates the fact that this is an important public health disease which merits a review of the implemented policies and activities and the generation of an intervention strategy which will ensure the commitment and participation of all the actors in the General Health and Social Security System:

- The Ministry of Health, following up on the compliance with the established regulations for ensuring healthy and safe work environments in underground mining activities.
- The health insurance agencies, which should establish mechanisms for early detection and follow-up of patients with a pneumoconiosis diagnosis, especially now that Resolution 1477 of 2014 classifies coal miner pneumoconiosis as a direct work-related illness.
- The workers compensation insurance companies, which should strengthen health promotion and disease prevention programs aimed at the mining sector and verify the efficacy of consulting and support for its affiliated companies in the implementation of safety and health management systems at work.
- The business owners, in relation to the need to implement
  - intervention strategies which will reduce the concentrations of bituminous coal dust and silica dust in order to reduce the level of risk to which the workers are exposed;
  - specific training programs which, taking into account the educational level of the workers, ensure awareness of the risk of developing pulmonary diseases;
  - epidemiologic surveillance programs for early detection of pulmonary alterations and the implementation of control strategies to avoid disease progression;
- finally, the workers who are responsible for complying with the safety and health norms in the workplace, including compliance with procedural protocols and the proper use of personal protection elements.

## Figures and Tables

**Table 1:** Population and sample distribution by department.

Department	Population		Sample	
	Number of employers	Number of workers	Number of employers	Number of workers
Boyacá	305	8667	18	232
Cundinamarca	161	5711	11	215
Total	**466**	**14378**	**29**	**447**

**Table 2:** Distribution of sociodemographic characteristics by department.

	Cundinamarca		Boyacá		Total	
	Number	%	Number	%	Number	%
Age group						
20–29	10	4.7	35	15.1	45	10.1
30–39	49	22.8	71	30.6	120	26.8
40–49	73	34.0	75	32.4	148	33.1
50–59	70	32.5	43	18.5	113	25.3
60 or more	13	6.0	8	3.4	21	4.7
Total	**215**	**100.0**	**232**	**100.0**	**447**	**100.0**
Place of residence						
Rural	111	51.6	167	72.0	278	62.2
Urban	104	48.4	65	28.0	169	37.8
Total	215	100.0	232	100.0	447	100.0
Socioeconomic strata						
1	8	3.7	56	24.6	64	14.4
2	125	58.1	117	51.3	242	54.6
3	68	31.6	51	22.4	119	26.9
4	12	5.6	3	1.3	15	3.4
5	2	0.9	1	0.4	3	0.7
Total	**215**	**99.9**	228^**∗**^	**100.0**	443^**∗**^	**100.0**
Educational level						
None	4	1.9	2	0.9	6	1.3
Incomplete elementary	83	38.6	77	33.2	160	35.8
Complete elementary	79	36.7	87	37.5	166	37.2
Incomplete secondary	29	13.5	32	13.8	61	13.6
Complete secondary	17	7.9	29	12.5	46	10.4
Incomplete technical training	0	0.0	1	0.4	1	0.2
Complete technical degree	1	0.5	4	1.7	5	1.1
Complete college	2	0.9	0	0.0	2	0.4
Total	**215**	**100.0**	**232**	**100.0**	**447**	**100.0**
Marital status						
Single	26	12.1	32	13.8	58	13.0
Married	81	37.7	100	43.1	181	40.5
Living together	93	43.3	86	37.1	179	40.0
Separated	12	5.5	13	5.6	25	5.6
Widowed	3	1.4	1	0.4	4	0.9
Total	**215**	**100.0**	**232**	**100.0**	**447**	**100.0**

The “*∗*” means that 4 people didn't answer the question about socioeconomic strata.

**Table 3:** Distribution of parenchymal abnormalities by department.

	Cundinamarca		Boyacá	
	Number	%	Number	%
Small opacities				
p/p	8	8.79%	8	11.59%
p/q	7	7.69%	8	11.59%
p/t	0	0.00%	1	1.45%
q/p	14	15.38%	19	27.54%
q/q	32	35.16%	31	44.93%
q/r	13	14.29%	2	2.90%
r/q	8	8.79%	0	0.00%
r/r	8	8.79%	0	0.00%
r/t	1	1.10%	0	0.00%
Total	91	100.00%	69	100.00%
Large opacities				
A	1	1.10%	2	2.90%
B	0	0.00%	2	2.90%
C	0	0.00%	0	0.00%
No	90	98.90%	65	94.20%
Total	91	100.00%	69	100.00%

**Table 4:** Relationship between coal dust (bituminous coal) and silica concentration risk levels and pneumoconiosis by department.

	Cundinamarca		Boyacá	
	Number	%
Coal scale				
Low	4	26.7%	1	11.1%
Medium	10	76.9%	0	0.0%
High	37	43.0%	41	27.9%
Severe	29	43.9%	19	44.2%
Total	80	44.4%	61	29.3%
Silica scale				
Low	11	31.4%	8	34.8%
Medium	12	50.0%	0	0.0%
High	6	50.0%	3	15.8%
Severe	42	45.2%	20	21.3%
Critical	20	39.2%	38	41.8%
Total	91	42.3%	69	29.9%

**Table 5:** Logistic regression of pneumoconiosis, company size, level of risk due to exposure to bituminous coal, length of work history, and department.

	*B*	Sig.	OR	95% CI (OR)
Department				
Cundinamarca	−0.040	0.887	0.961	0.553–1.670
Boyacá			1.000	
Company size		0.019		
Large	0.111	0.748	1.118	0.567–2.203
Medium	0.833	0.007	2.301	1.260–4.201
Small			1.000	
Coal dust level of risk		0.227		
Severe	1.065	0.065	2.901	0.937–8.982
High	0.679	0.217	1.972	0.671–5.799
Medium	1.009	0.149	2.744	0.697–10.804
Low			1.000	
Number of years at work				
≥30 years	1.170	0.000	3.222	1.806–5.748
25–29.9	1.306	0.000	3.691	1.892–7.201
<25 years			1.000	
Smoking for more than one year				
Yes	0.392	0.092	1.479	0.938–2.334
No			1.000	
Constant	−2.183	0.000	0.113	

## Limitations

Although companies and selected workers were only linked to one of 13 insurance companies in the country, it is important to note that the research was conducted in the insurance company under the Ministry of Finance and Public Credit, which brings together the largest number of mining companies and miners in Colombia. It is a cross-sectional study, so it is not possible determine the coal miners' accumulated exposure to coal dust over time. Finally, only one reader made the classification of chest radiographs using the ILO criteria, so the results may reflect trends in classification of the individual readers.
